# Supplementary material for: Modeling glioblastoma heterogeneity as a dynamic network of cell states
Source: Mol Syst Biol. 2021 Sep 16;17(9):e10105. doi: 10.15252/msb.202010105 (PMC8444284; doi:10.15252/msb.202010105)
Supplement: Supplementary file 5 — Source Data for Figure 3 [file MSB-17-e10105-s001.zip › Figure3A_sourcedata/GSEA_3065/nosigdb_state2.GseaPreranked.1622718419215/FELDMAN_2020_S-G2.html]

Details for gene set FELDMAN\_2020\_S-G2[GSEA]

|  || Dataset | state2 |
| Phenotype | NoPhenotypeAvailable |
| Upregulated in class | na\_pos |
| GeneSet | FELDMAN\_2020\_S-G2 |
| Enrichment Score (ES) | 0.796391 |
| Normalized Enrichment Score (NES) | 3.001823 |
| Nominal p-value | 0.0 |
| FDR q-value | 0.0 |
| FWER p-Value | 0.0 |
Table: GSEA Results Summary

  

Fig 1: Enrichment plot: FELDMAN\_2020\_S-G2      
 Profile of the Running ES Score & Positions of GeneSet Members on the Rank Ordered List

  

| PROBE | GENE SYMBOL | GENE\_TITLE | RANK IN GENE LIST | RANK METRIC SCORE | RUNNING ES | CORE ENRICHMENT || 1 | HIST1H4C |  |  | 0 | 1.309 | 0.0137 | Yes |
| 2 | HIST1H1B |  |  | 1 | 0.902 | 0.0231 | Yes |
| 3 | HIST1H1D |  |  | 3 | 0.842 | 0.0318 | Yes |
| 4 | HMGB2 |  |  | 4 | 0.823 | 0.0404 | Yes |
| 5 | TYMS |  |  | 5 | 0.792 | 0.0487 | Yes |
| 6 | CENPU |  |  | 6 | 0.763 | 0.0567 | Yes |
| 7 | H2AFZ |  |  | 8 | 0.735 | 0.0643 | Yes |
| 8 | UBE2C |  |  | 9 | 0.704 | 0.0716 | Yes |
| 9 | H2AFX |  |  | 10 | 0.675 | 0.0787 | Yes |
| 10 | DHFR |  |  | 11 | 0.644 | 0.0854 | Yes |
| 11 | UBE2T |  |  | 12 | 0.643 | 0.0921 | Yes |
| 12 | MCM7 |  |  | 13 | 0.626 | 0.0987 | Yes |
| 13 | MYBL2 |  |  | 14 | 0.600 | 0.1050 | Yes |
| 14 | TOP2A |  |  | 15 | 0.596 | 0.1112 | Yes |
| 15 | PCNA |  |  | 16 | 0.595 | 0.1174 | Yes |
| 16 | MKI67 |  |  | 17 | 0.590 | 0.1236 | Yes |
| 17 | CDK1 |  |  | 18 | 0.577 | 0.1296 | Yes |
| 18 | UBE2S |  |  | 19 | 0.574 | 0.1356 | Yes |
| 19 | MAD2L1 |  |  | 20 | 0.563 | 0.1415 | Yes |
| 20 | ATAD2 |  |  | 21 | 0.563 | 0.1474 | Yes |
| 21 | RRM2 |  |  | 22 | 0.556 | 0.1532 | Yes |
| 22 | CKS1B |  |  | 23 | 0.554 | 0.1590 | Yes |
| 23 | DUT |  |  | 24 | 0.552 | 0.1648 | Yes |
| 24 | HMGB1 |  |  | 25 | 0.549 | 0.1705 | Yes |
| 25 | NUDT1 |  |  | 26 | 0.541 | 0.1762 | Yes |
| 26 | CLSPN |  |  | 27 | 0.536 | 0.1818 | Yes |
| 27 | CKS2 |  |  | 28 | 0.532 | 0.1873 | Yes |
| 28 | TPX2 |  |  | 29 | 0.531 | 0.1929 | Yes |
| 29 | ESCO2 |  |  | 30 | 0.530 | 0.1984 | Yes |
| 30 | DEK |  |  | 31 | 0.526 | 0.2039 | Yes |
| 31 | STMN1 |  |  | 33 | 0.520 | 0.2092 | Yes |
| 32 | CENPF |  |  | 34 | 0.518 | 0.2147 | Yes |
| 33 | HMGN2 |  |  | 35 | 0.512 | 0.2200 | Yes |
| 34 | RPA3 |  |  | 36 | 0.511 | 0.2254 | Yes |
| 35 | GINS2 |  |  | 37 | 0.506 | 0.2307 | Yes |
| 36 | HIST1H1C |  |  | 38 | 0.498 | 0.2359 | Yes |
| 37 | SMC2 |  |  | 39 | 0.493 | 0.2410 | Yes |
| 38 | ORC6 |  |  | 40 | 0.493 | 0.2462 | Yes |
| 39 | DBF4 |  |  | 41 | 0.492 | 0.2513 | Yes |
| 40 | SMC4 |  |  | 42 | 0.489 | 0.2564 | Yes |
| 41 | TMPO |  |  | 43 | 0.489 | 0.2615 | Yes |
| 42 | CENPK |  |  | 44 | 0.488 | 0.2666 | Yes |
| 43 | ZWINT |  |  | 45 | 0.488 | 0.2717 | Yes |
| 44 | PBK |  |  | 46 | 0.484 | 0.2768 | Yes |
| 45 | NUSAP1 |  |  | 47 | 0.483 | 0.2819 | Yes |
| 46 | ANP32B |  |  | 48 | 0.474 | 0.2868 | Yes |
| 47 | PTMA |  |  | 49 | 0.474 | 0.2918 | Yes |
| 48 | KPNA2 |  |  | 50 | 0.472 | 0.2967 | Yes |
| 49 | MCM3 |  |  | 51 | 0.464 | 0.3015 | Yes |
| 50 | TUBA1B |  |  | 52 | 0.462 | 0.3064 | Yes |
| 51 | RFC2 |  |  | 53 | 0.460 | 0.3112 | Yes |
| 52 | CENPM |  |  | 54 | 0.460 | 0.3160 | Yes |
| 53 | TK1 |  |  | 56 | 0.456 | 0.3207 | Yes |
| 54 | NCAPG |  |  | 60 | 0.445 | 0.3250 | Yes |
| 55 | BIRC5 |  |  | 61 | 0.443 | 0.3296 | Yes |
| 56 | SIVA1 |  |  | 62 | 0.439 | 0.3342 | Yes |
| 57 | PTTG1 |  |  | 63 | 0.439 | 0.3388 | Yes |
| 58 | GMNN |  |  | 64 | 0.435 | 0.3433 | Yes |
| 59 | RANBP1 |  |  | 65 | 0.430 | 0.3478 | Yes |
| 60 | HIST1H1E |  |  | 66 | 0.429 | 0.3523 | Yes |
| 61 | FBXO5 |  |  | 67 | 0.420 | 0.3567 | Yes |
| 62 | LMNB1 |  |  | 69 | 0.417 | 0.3610 | Yes |
| 63 | CENPH |  |  | 71 | 0.412 | 0.3652 | Yes |
| 64 | RRM1 |  |  | 72 | 0.412 | 0.3695 | Yes |
| 65 | LSM4 |  |  | 73 | 0.410 | 0.3738 | Yes |
| 66 | SNRPD1 |  |  | 74 | 0.408 | 0.3780 | Yes |
| 67 | ASF1B |  |  | 76 | 0.407 | 0.3822 | Yes |
| 68 | TUBB |  |  | 81 | 0.397 | 0.3859 | Yes |
| 69 | H2AFV |  |  | 82 | 0.397 | 0.3900 | Yes |
| 70 | KIF4A |  |  | 84 | 0.393 | 0.3940 | Yes |
| 71 | CENPN |  |  | 87 | 0.390 | 0.3979 | Yes |
| 72 | HNRNPD |  |  | 88 | 0.390 | 0.4020 | Yes |
| 73 | GTSE1 |  |  | 89 | 0.389 | 0.4060 | Yes |
| 74 | RAD51AP1 |  |  | 90 | 0.388 | 0.4101 | Yes |
| 75 | DTYMK |  |  | 94 | 0.379 | 0.4137 | Yes |
| 76 | CDC45 |  |  | 95 | 0.377 | 0.4176 | Yes |
| 77 | MND1 |  |  | 96 | 0.376 | 0.4216 | Yes |
| 78 | RAN |  |  | 98 | 0.375 | 0.4254 | Yes |
| 79 | GGCT |  |  | 102 | 0.373 | 0.4290 | Yes |
| 80 | DNAJC9 |  |  | 103 | 0.373 | 0.4328 | Yes |
| 81 | SPC25 |  |  | 104 | 0.372 | 0.4367 | Yes |
| 82 | CENPE |  |  | 105 | 0.372 | 0.4406 | Yes |
| 83 | FEN1 |  |  | 107 | 0.371 | 0.4444 | Yes |
| 84 | CACYBP |  |  | 109 | 0.366 | 0.4481 | Yes |
| 85 | RHEB |  |  | 110 | 0.362 | 0.4519 | Yes |
| 86 | ANLN |  |  | 111 | 0.358 | 0.4556 | Yes |
| 87 | HNRNPAB |  |  | 113 | 0.357 | 0.4593 | Yes |
| 88 | NDC80 |  |  | 115 | 0.354 | 0.4628 | Yes |
| 89 | CENPV |  |  | 116 | 0.353 | 0.4665 | Yes |
| 90 | KIF23 |  |  | 117 | 0.351 | 0.4702 | Yes |
| 91 | VRK1 |  |  | 118 | 0.350 | 0.4739 | Yes |
| 92 | POP7 |  |  | 119 | 0.347 | 0.4775 | Yes |
| 93 | DNMT1 |  |  | 121 | 0.345 | 0.4810 | Yes |
| 94 | ASPM |  |  | 122 | 0.342 | 0.4846 | Yes |
| 95 | TUBB4B |  |  | 125 | 0.340 | 0.4879 | Yes |
| 96 | RFC4 |  |  | 126 | 0.339 | 0.4915 | Yes |
| 97 | CCDC34 |  |  | 127 | 0.339 | 0.4950 | Yes |
| 98 | PTGES3 |  |  | 128 | 0.336 | 0.4985 | Yes |
| 99 | TACC3 |  |  | 130 | 0.334 | 0.5019 | Yes |
| 100 | SNRPB |  |  | 132 | 0.332 | 0.5053 | Yes |
| 101 | MELK |  |  | 133 | 0.332 | 0.5087 | Yes |
| 102 | CCNA2 |  |  | 135 | 0.331 | 0.5121 | Yes |
| 103 | PA2G4 |  |  | 137 | 0.329 | 0.5154 | Yes |
| 104 | USP1 |  |  | 138 | 0.329 | 0.5189 | Yes |
| 105 | SET |  |  | 139 | 0.328 | 0.5223 | Yes |
| 106 | SRSF3 |  |  | 140 | 0.327 | 0.5257 | Yes |
| 107 | RBBP8 |  |  | 141 | 0.325 | 0.5291 | Yes |
| 108 | MIS18A |  |  | 142 | 0.322 | 0.5325 | Yes |
| 109 | CALM2 |  |  | 143 | 0.322 | 0.5358 | Yes |
| 110 | SNRPG |  |  | 144 | 0.322 | 0.5392 | Yes |
| 111 | ARHGAP11A |  |  | 145 | 0.321 | 0.5425 | Yes |
| 112 | NCL |  |  | 146 | 0.316 | 0.5459 | Yes |
| 113 | CDCA2 |  |  | 150 | 0.309 | 0.5488 | Yes |
| 114 | CKAP2 |  |  | 151 | 0.308 | 0.5520 | Yes |
| 115 | KIF22 |  |  | 154 | 0.306 | 0.5550 | Yes |
| 116 | SRSF7 |  |  | 155 | 0.306 | 0.5582 | Yes |
| 117 | NASP |  |  | 156 | 0.306 | 0.5614 | Yes |
| 118 | SNRPE |  |  | 157 | 0.306 | 0.5646 | Yes |
| 119 | CEP55 |  |  | 158 | 0.306 | 0.5678 | Yes |
| 120 | PSMC3IP |  |  | 159 | 0.305 | 0.5710 | Yes |
| 121 | CDCA3 |  |  | 160 | 0.304 | 0.5741 | Yes |
| 122 | HELLS |  |  | 161 | 0.304 | 0.5773 | Yes |
| 123 | TMEM106C |  |  | 162 | 0.303 | 0.5805 | Yes |
| 124 | AURKB |  |  | 163 | 0.303 | 0.5836 | Yes |
| 125 | CHCHD2 |  |  | 165 | 0.302 | 0.5867 | Yes |
| 126 | SLC25A5 |  |  | 168 | 0.299 | 0.5896 | Yes |
| 127 | CMC2 |  |  | 170 | 0.297 | 0.5926 | Yes |
| 128 | DCTPP1 |  |  | 172 | 0.296 | 0.5956 | Yes |
| 129 | BTG3 |  |  | 173 | 0.295 | 0.5987 | Yes |
| 130 | MZT1 |  |  | 174 | 0.295 | 0.6018 | Yes |
| 131 | PSIP1 |  |  | 175 | 0.294 | 0.6048 | Yes |
| 132 | HSPD1 |  |  | 176 | 0.294 | 0.6079 | Yes |
| 133 | CARHSP1 |  |  | 178 | 0.294 | 0.6109 | Yes |
| 134 | HMGN1 |  |  | 182 | 0.293 | 0.6136 | Yes |
| 135 | CDCA4 |  |  | 184 | 0.292 | 0.6165 | Yes |
| 136 | PDCD5 |  |  | 186 | 0.291 | 0.6195 | Yes |
| 137 | PAICS |  |  | 187 | 0.291 | 0.6225 | Yes |
| 138 | NUF2 |  |  | 188 | 0.287 | 0.6255 | Yes |
| 139 | HAUS1 |  |  | 192 | 0.285 | 0.6282 | Yes |
| 140 | PARP1 |  |  | 194 | 0.284 | 0.6310 | Yes |
| 141 | HSPA8 |  |  | 195 | 0.283 | 0.6340 | Yes |
| 142 | DEPDC1 |  |  | 196 | 0.283 | 0.6369 | Yes |
| 143 | SMC3 |  |  | 197 | 0.283 | 0.6399 | Yes |
| 144 | MAGOHB |  |  | 199 | 0.282 | 0.6427 | Yes |
| 145 | HNRNPF |  |  | 205 | 0.279 | 0.6451 | Yes |
| 146 | TEX30 |  |  | 206 | 0.279 | 0.6480 | Yes |
| 147 | CYCS |  |  | 207 | 0.279 | 0.6509 | Yes |
| 148 | KIF20B |  |  | 208 | 0.278 | 0.6539 | Yes |
| 149 | HJURP |  |  | 209 | 0.277 | 0.6568 | Yes |
| 150 | HSPB11 |  |  | 210 | 0.277 | 0.6597 | Yes |
| 151 | HSPE1 |  |  | 211 | 0.276 | 0.6625 | Yes |
| 152 | SRSF2 |  |  | 213 | 0.275 | 0.6653 | Yes |
| 153 | C19orf48 |  |  | 215 | 0.274 | 0.6681 | Yes |
| 154 | DSN1 |  |  | 216 | 0.273 | 0.6709 | Yes |
| 155 | HNRNPDL |  |  | 220 | 0.271 | 0.6734 | Yes |
| 156 | YWHAH |  |  | 221 | 0.271 | 0.6762 | Yes |
| 157 | ATAD5 |  |  | 222 | 0.269 | 0.6791 | Yes |
| 158 | SHCBP1 |  |  | 231 | 0.262 | 0.6809 | Yes |
| 159 | EIF4EBP1 |  |  | 232 | 0.261 | 0.6836 | Yes |
| 160 | WDR34 |  |  | 235 | 0.260 | 0.6861 | Yes |
| 161 | CENPA |  |  | 238 | 0.259 | 0.6886 | Yes |
| 162 | PGP |  |  | 244 | 0.254 | 0.6907 | Yes |
| 163 | SMC1A |  |  | 249 | 0.253 | 0.6929 | Yes |
| 164 | CKLF |  |  | 254 | 0.249 | 0.6951 | Yes |
| 165 | MIS18BP1 |  |  | 257 | 0.246 | 0.6975 | Yes |
| 166 | MRPL18 |  |  | 258 | 0.246 | 0.7000 | Yes |
| 167 | TUBG1 |  |  | 262 | 0.246 | 0.7023 | Yes |
| 168 | SAC3D1 |  |  | 263 | 0.246 | 0.7048 | Yes |
| 169 | RAD23A |  |  | 264 | 0.245 | 0.7074 | Yes |
| 170 | HIST2H2AC |  |  | 267 | 0.244 | 0.7097 | Yes |
| 171 | UBE2I |  |  | 273 | 0.242 | 0.7117 | Yes |
| 172 | PSMC3 |  |  | 275 | 0.242 | 0.7141 | Yes |
| 173 | CHCHD3 |  |  | 277 | 0.242 | 0.7166 | Yes |
| 174 | SAE1 |  |  | 278 | 0.242 | 0.7191 | Yes |
| 175 | CMSS1 |  |  | 281 | 0.241 | 0.7214 | Yes |
| 176 | CEP78 |  |  | 283 | 0.239 | 0.7238 | Yes |
| 177 | TRIP13 |  |  | 286 | 0.237 | 0.7260 | Yes |
| 178 | RBM17 |  |  | 291 | 0.236 | 0.7281 | Yes |
| 179 | PIN1 |  |  | 296 | 0.234 | 0.7301 | Yes |
| 180 | RFC5 |  |  | 297 | 0.233 | 0.7325 | Yes |
| 181 | PRELID1 |  |  | 300 | 0.231 | 0.7347 | Yes |
| 182 | POLR3K |  |  | 309 | 0.229 | 0.7362 | Yes |
| 183 | BUB3 |  |  | 311 | 0.228 | 0.7385 | Yes |
| 184 | C12orf75 |  |  | 316 | 0.227 | 0.7404 | Yes |
| 185 | TCF19 |  |  | 318 | 0.226 | 0.7427 | Yes |
| 186 | SSRP1 |  |  | 319 | 0.226 | 0.7451 | Yes |
| 187 | DIAPH3 |  |  | 321 | 0.226 | 0.7473 | Yes |
| 188 | NXT1 |  |  | 326 | 0.224 | 0.7492 | Yes |
| 189 | CKAP2L |  |  | 327 | 0.224 | 0.7516 | Yes |
| 190 | MXD3 |  |  | 328 | 0.224 | 0.7539 | Yes |
| 191 | MRPL17 |  |  | 344 | 0.218 | 0.7545 | Yes |
| 192 | HIRIP3 |  |  | 347 | 0.217 | 0.7566 | Yes |
| 193 | KPNB1 |  |  | 350 | 0.216 | 0.7586 | Yes |
| 194 | EIF1AX |  |  | 356 | 0.215 | 0.7603 | Yes |
| 195 | SNRPA1 |  |  | 361 | 0.214 | 0.7621 | Yes |
| 196 | CENPW |  |  | 373 | 0.211 | 0.7631 | Yes |
| 197 | ZWILCH |  |  | 374 | 0.210 | 0.7653 | Yes |
| 198 | HAT1 |  |  | 381 | 0.209 | 0.7668 | Yes |
| 199 | RNPS1 |  |  | 410 | 0.198 | 0.7658 | Yes |
| 200 | BRCA2 |  |  | 427 | 0.193 | 0.7661 | Yes |
| 201 | PSMG1 |  |  | 428 | 0.193 | 0.7681 | Yes |
| 202 | PXMP2 |  |  | 430 | 0.193 | 0.7700 | Yes |
| 203 | COPS3 |  |  | 437 | 0.192 | 0.7714 | Yes |
| 204 | EXOSC8 |  |  | 456 | 0.189 | 0.7714 | Yes |
| 205 | SPDL1 |  |  | 459 | 0.189 | 0.7731 | Yes |
| 206 | RTKN2 |  |  | 463 | 0.188 | 0.7748 | Yes |
| 207 | DCK |  |  | 465 | 0.187 | 0.7766 | Yes |
| 208 | DERA |  |  | 471 | 0.185 | 0.7780 | Yes |
| 209 | SNRNP40 |  |  | 480 | 0.184 | 0.7791 | Yes |
| 210 | IER2 |  |  | 482 | 0.183 | 0.7809 | Yes |
| 211 | CDKN2D |  |  | 499 | 0.182 | 0.7810 | Yes |
| 212 | RNASEH2B |  |  | 507 | 0.179 | 0.7821 | Yes |
| 213 | PRC1 |  |  | 514 | 0.178 | 0.7833 | Yes |
| 214 | TRA2B |  |  | 515 | 0.178 | 0.7852 | Yes |
| 215 | LRR1 |  |  | 516 | 0.178 | 0.7870 | Yes |
| 216 | SNRNP25 |  |  | 525 | 0.177 | 0.7880 | Yes |
| 217 | SUPT16H |  |  | 529 | 0.176 | 0.7895 | Yes |
| 218 | CTNNAL1 |  |  | 532 | 0.176 | 0.7911 | Yes |
| 219 | TOMM40 |  |  | 560 | 0.171 | 0.7900 | Yes |
| 220 | HPRT1 |  |  | 573 | 0.168 | 0.7904 | Yes |
| 221 | KIFC1 |  |  | 577 | 0.167 | 0.7918 | Yes |
| 222 | RMI2 |  |  | 579 | 0.166 | 0.7934 | Yes |
| 223 | BRIX1 |  |  | 588 | 0.165 | 0.7943 | Yes |
| 224 | HSP90AA1 |  |  | 600 | 0.164 | 0.7948 | Yes |
| 225 | PFDN2 |  |  | 602 | 0.163 | 0.7964 | Yes |
| 226 | TUBB6 |  |  | 622 | 0.160 | 0.7960 | No |
| 227 | S100B |  |  | 688 | 0.151 | 0.7904 | No |
| 228 | EIF5 |  |  | 699 | 0.150 | 0.7909 | No |
| 229 | FBL |  |  | 738 | 0.146 | 0.7883 | No |
| 230 | SKA3 |  |  | 790 | 0.139 | 0.7841 | No |
| 231 | FABP7 |  |  | 810 | 0.137 | 0.7835 | No |
| 232 | SMS |  |  | 846 | 0.133 | 0.7810 | No |
| 233 | SYNE2 |  |  | 853 | 0.132 | 0.7817 | No |
| 234 | TFDP1 |  |  | 874 | 0.130 | 0.7809 | No |
| 235 | PARP2 |  |  | 886 | 0.129 | 0.7810 | No |
| 236 | DONSON |  |  | 929 | 0.125 | 0.7777 | No |
| 237 | EIF2S1 |  |  | 1051 | 0.115 | 0.7657 | No |
| 238 | ACYP1 |  |  | 1109 | 0.111 | 0.7606 | No |
| 239 | RPL22L1 |  |  | 1112 | 0.111 | 0.7615 | No |
| 240 | ARL6IP6 |  |  | 1134 | 0.109 | 0.7603 | No |
| 241 | CISD2 |  |  | 1219 | 0.103 | 0.7522 | No |
| 242 | SOX9 |  |  | 1466 | 0.088 | 0.7261 | No |
| 243 | ST13 |  |  | 1545 | 0.084 | 0.7185 | No |
| 244 | RNASEH2A |  |  | 1579 | 0.082 | 0.7157 | No |
| 245 | RRS1 |  |  | 1618 | 0.081 | 0.7124 | No |
| 246 | HIST1H2AH |  |  | 1672 | 0.078 | 0.7074 | No |
| 247 | PRDX1 |  |  | 1763 | 0.074 | 0.6983 | No |
| 248 | PFDN5 |  |  | 1770 | 0.074 | 0.6984 | No |
| 249 | HOXB2 |  |  | 1771 | 0.074 | 0.6991 | No |
| 250 | TUBA1A |  |  | 2004 | 0.064 | 0.6744 | No |
| 251 | GGH |  |  | 2149 | 0.059 | 0.6592 | No |
| 252 | PRKDC |  |  | 2154 | 0.059 | 0.6593 | No |
| 253 | NUDT15 |  |  | 2188 | 0.058 | 0.6563 | No |
| 254 | ATP6V1G1 |  |  | 2201 | 0.057 | 0.6556 | No |
| 255 | RPL34 |  |  | 2224 | 0.056 | 0.6538 | No |
| 256 | RPL39L |  |  | 2249 | 0.055 | 0.6517 | No |
| 257 | C4orf3 |  |  | 2337 | 0.052 | 0.6427 | No |
| 258 | COMMD6 |  |  | 2534 | 0.047 | 0.6217 | No |
| 259 | FOSL1 |  |  | 2584 | 0.045 | 0.6168 | No |
| 260 | TMEM237 |  |  | 2862 | 0.037 | 0.5868 | No |
| 261 | SDCBP |  |  | 3058 | 0.033 | 0.5657 | No |
| 262 | RGCC |  |  | 3143 | 0.031 | 0.5568 | No |
| 263 | RAB13 |  |  | 3928 | 0.017 | 0.4710 | No |
| 264 | HMGA1 |  |  | 4135 | 0.013 | 0.4485 | No |
| 265 | MLLT11 |  |  | 4607 | 0.006 | 0.3968 | No |
| 266 | PLP2 |  |  | 5612 | -0.010 | 0.2868 | No |
| 267 | PMAIP1 |  |  | 5617 | -0.010 | 0.2864 | No |
| 268 | UPP1 |  |  | 6249 | -0.021 | 0.2174 | No |
| 269 | AKAP12 |  |  | 6471 | -0.025 | 0.1934 | No |
| 270 | WDR83OS |  |  | 6589 | -0.027 | 0.1808 | No |
| 271 | EEF2 |  |  | 6630 | -0.028 | 0.1767 | No |
| 272 | EEF1A1 |  |  | 6690 | -0.029 | 0.1706 | No |
| 273 | BAMBI |  |  | 6859 | -0.033 | 0.1525 | No |
| 274 | PHLDA1 |  |  | 6867 | -0.033 | 0.1520 | No |
| 275 | LYPD1 |  |  | 6876 | -0.033 | 0.1515 | No |
| 276 | BEX4 |  |  | 6903 | -0.034 | 0.1490 | No |
| 277 | EPS8L1 |  |  | 6940 | -0.035 | 0.1454 | No |
| 278 | KLF6 |  |  | 7010 | -0.037 | 0.1382 | No |
| 279 | RPS27L |  |  | 7139 | -0.040 | 0.1246 | No |
| 280 | NETO2 |  |  | 7418 | -0.048 | 0.0946 | No |
| 281 | FTL |  |  | 7693 | -0.056 | 0.0651 | No |
| 282 | ZFAND6 |  |  | 7733 | -0.057 | 0.0614 | No |
| 283 | HES4 |  |  | 7819 | -0.060 | 0.0527 | No |
| 284 | YPEL5 |  |  | 7885 | -0.062 | 0.0463 | No |
| 285 | TNFRSF12A |  |  | 7931 | -0.064 | 0.0420 | No |
| 286 | PLAU |  |  | 8131 | -0.073 | 0.0209 | No |
| 287 | BTG1 |  |  | 8156 | -0.075 | 0.0191 | No |
| 288 | RNF19A |  |  | 8467 | -0.093 | -0.0140 | No |
| 289 | MAGED2 |  |  | 8572 | -0.101 | -0.0243 | No |
| 290 | PLD3 |  |  | 8576 | -0.102 | -0.0236 | No |
| 291 | DDIT4 |  |  | 8601 | -0.103 | -0.0252 | No |
| 292 | MAP3K7CL |  |  | 8672 | -0.109 | -0.0317 | No |
| 293 | IFITM3 |  |  | 8720 | -0.113 | -0.0357 | No |
| 294 | ENC1 |  |  | 8759 | -0.117 | -0.0386 | No |
| 295 | TMEM158 |  |  | 8846 | -0.128 | -0.0467 | No |
| 296 | CNPY2 |  |  | 8913 | -0.137 | -0.0525 | No |
| 297 | PLP1 |  |  | 8931 | -0.139 | -0.0530 | No |
| 298 | TTYH1 |  |  | 9029 | -0.154 | -0.0620 | No |
| 299 | SOX4 |  |  | 9063 | -0.160 | -0.0639 | No |
| 300 | EIF4A2 |  |  | 9067 | -0.160 | -0.0626 | No |
| 301 | BEX1 |  |  | 9079 | -0.162 | -0.0621 | No |
| 302 | TMEM59 |  |  | 9085 | -0.163 | -0.0610 | No |
| 303 | SCD5 |  |  | 9133 | -0.174 | -0.0643 | No |
| 304 | NDFIP1 |  |  | 9140 | -0.175 | -0.0631 | No |
| 305 | PLAT |  |  | 9145 | -0.176 | -0.0617 | No |
| 306 | PRCP |  |  | 9153 | -0.178 | -0.0606 | No |
| 307 | SCRG1 |  |  | 9200 | -0.191 | -0.0637 | No |
| 308 | ID3 |  |  | 9252 | -0.214 | -0.0670 | No |
| 309 | CDH2 |  |  | 9253 | -0.215 | -0.0648 | No |
| 310 | SESN3 |  |  | 9266 | -0.219 | -0.0638 | No |
| 311 | RABAC1 |  |  | 9270 | -0.220 | -0.0618 | No |
| 312 | SAT1 |  |  | 9279 | -0.224 | -0.0604 | No |
| 313 | CDKN1A |  |  | 9301 | -0.230 | -0.0603 | No |
| 314 | PCSK1N |  |  | 9321 | -0.247 | -0.0598 | No |
| 315 | TM7SF2 |  |  | 9330 | -0.252 | -0.0580 | No |
| 316 | PTPRZ1 |  |  | 9351 | -0.272 | -0.0574 | No |
| 317 | PSAP |  |  | 9363 | -0.294 | -0.0555 | No |
| 318 | LAPTM4A |  |  | 9368 | -0.299 | -0.0528 | No |
| 319 | GPM6A |  |  | 9381 | -0.324 | -0.0508 | No |
| 320 | SPARC |  |  | 9384 | -0.332 | -0.0475 | No |
| 321 | MIAT |  |  | 9386 | -0.332 | -0.0441 | No |
| 322 | GPM6B |  |  | 9389 | -0.344 | -0.0408 | No |
| 323 | ITM2B |  |  | 9399 | -0.371 | -0.0379 | No |
| 324 | CTGF |  |  | 9400 | -0.375 | -0.0340 | No |
| 325 | ARL6IP5 |  |  | 9420 | -0.467 | -0.0312 | No |
| 326 | IGFBP3 |  |  | 9424 | -0.502 | -0.0262 | No |
| 327 | CLU |  |  | 9430 | -0.547 | -0.0211 | No |
| 328 | DCBLD2 |  |  | 9431 | -0.558 | -0.0152 | No |
| 329 | MALAT1 |  |  | 9436 | -0.735 | -0.0080 | No |
| 330 | IGFBP5 |  |  | 9438 | -0.797 | 0.0002 | No |
Table: GSEA details [plain text format]

  

Fig 2: FELDMAN\_2020\_S-G2: Random ES distribution      
 Gene set null distribution of ES for **FELDMAN\_2020\_S-G2**
